# Establishing and sustaining mosquito colonies: insights into morphology, bionomics, and advances in the rearing of *Anopheles*, *Culex*, and *Aedes*

**DOI:** 10.3389/finsc.2026.1766919

**Published:** 2026-04-17

**Authors:** Irrusappan Hari, Prajwal Gaikwad, Sanket Kumar Ray, Jose Antony Jenish, Tharani Priya P, Kalichamy Alagarasu, Balasubramanian Rathinam, Balachandar Vellingiri, Devakumar Dinesh, Kalpana Baruah

**Affiliations:** 1 ICMR-National Institute of Virology, Pune, Maharashtra, India; 2 Academy of Scientific and Innovative Research, Ghaziabad, Uttar Pradesh, India; 3 ICMR-National Institute for Vector Control Research, Puducherry, India; 4 Bharathidasan University, Tiruchirappalli, Tamil Nadu, India; 5 Central University of Punjab, Bathinda, Punjab, India; 6 National Center for Vector Borne Diseases Control, Delhi, India

**Keywords:** *Aedes*, *Anopheles*, colony maintenance, *Culex*, Diptera, laboratory rearing, mosquito bionomics, mosquito morphology

## Abstract

Mosquitoes of the genera *Anopheles*, *Culex*, and *Aedes* are of major medical importance as vectors of malaria, filariasis, Japanese encephalitis, dengue, chikungunya, and Zika. Understanding their morphology and bionomics is fundamental for vector biology, ecological studies, and the design of effective control strategies. Laboratory colonies of mosquitoes serve as an indispensable resource for investigating mosquito genetics, physiology, and vector–pathogen interactions, while also enabling the evaluation of new interventions such as Wolbachia-based control and the sterile insect technique. This review synthesizes current knowledge on the morphology and bionomics of key mosquito vectors and outlines advances in colony establishment, rearing, and maintenance. Particular attention is given to larval and adult diet, environmental conditions, oviposition, blood-feeding methods, and strategies to minimize inbreeding and maintain microbial balance in colonies. Challenges including genetic drift, colony adaptation, and maintenance of representative field traits are discussed alongside emerging solutions. By integrating classical entomological knowledge with recent innovations in rearing technology, this review highlights the central role of sustainable mosquito colonies in strengthening basic research and supporting applied approaches for vector-borne disease control.

## 1 Introduction

Mosquitoes are one of the most studied insects on the planet. They merit this kind of attention due to their involvement in the transmission of viral and protozoan disease and annoying biting behavior. Mosquitoes belong to the family Culicidae, comprising approximately 3,600 species worldwide. These insects exhibit diverse ecological and physiological adaptations that allow them to thrive in a wide range of environments from tropical and subtropical regions to elevations as high as 3,500 meters and depths of 1,250 meters below sea level (1, 2). Their adaptability underscores their potential to serve as vectors in varied ecological settings. Many mosquito species are important in public health because of their blood-feeding behavior that helps in transmitting pathogens (1–3). Public health emergencies caused by mosquito-borne diseases in the present and past made the public health and veterinary entomology field very important (4, 5). Well-known mosquito-borne diseases are malaria, dengue, Zika, filariasis, chikungunya, Japanese encephalitis, West Nile fever and yellow fever (3). *Anopheles*, *Culex*, and *Aedes* are the three major genera of mosquitoes that transmit several diseases to mankind. The *Anopheles* mosquito is a key genus implicated in malaria transmission (6, 7). The *Culex* mosquito is a widespread genus found worldwide, except for extreme northern and southern temperate regions and is involved in the spread of diseases like lymphatic filariasis and Japanese encephalitis. *Aedes* mosquitoes are involved in transmitting diseases like Zika, dengue, chikungunya and yellow fever.

Vector-borne pathogens are transmitted through several mechanisms. Biological transmission involves pathogen development or replication within the vector before transmission, whereas mechanical transmission is passive, with no replication (e.g., contaminated mouthparts). Further, developmental transmission involves only parasite development in the vector; cyclo-developmental involves development and morphological changes without multiplication; and cyclo-propagative transmission includes both development and multiplication within the vector. These classifications are critical for understanding vector competence and disease epidemiology (8, 9).

To transmit a disease, the mosquito needs to be competent enough to acquire, propagate, and transmit the pathogen, which is known as vector competence of mosquitoes (10, 11). Understanding the biology of mosquitoes and the pathogens they transmit is important for controlling and preventing the diseases they transmit (12, 13). Research involving mosquitoes and mosquito-borne diseases requires standard colonies of mosquitoes with required containment facilities which follow biosafety norms. The need for *Aedes* mosquito laboratory colonization and pure strain maintenance is also equally important to conduct any research studies (14–16).

The process of reproducing and keeping particular organisms, like insects or other species, in a controlled laboratory setting is referred to as laboratory colonization (17–19). Laboratory colonization and mass rearing of mosquitoes is particularly helpful as it provides a consistent and controlled source of mosquitoes for research, enabling standardized experiments and reliable data (20–22). Studies on vector biology, bio-ecology, vector-pathogen interactions, pesticide susceptibility evaluation of repellents and other control measures require the establishment and maintenance of mosquito colonies in laboratory settings (20, 23, 24). The mosquitoes’ original gene pool, physiological traits, and behavioral traits being studied must therefore be preserved to the greatest extent feasible (21). Establishing healthy and standardized mosquito colonies requires protocol generation and implementation, detailed attention to mosquito life stages, and a thorough understanding of factors that influence mosquito fitness, which includes temperature, humidity, nutrient quality, quantity, availability, blood-feeding behavior and oviposition requirements (18, 21, 22).

This review analyses the current methodologies and advancements in laboratory colonization, mass rearing, and the morphology and bionomics. By addressing critical questions such as the techniques required for mosquito colonization, the role of mass rearing in advancing vector biology research, we aim to synthesize available knowledge while identifying crucial gaps. This review seeks to provide a comprehensive resource for researchers, offering insights to refine laboratory-rearing practices and support innovative approaches in mosquito research and vector control.

Established laboratory colonies of medically important mosquitoes provide standardized model systems that have been instrumental in advancing vector biology and vector control research. Controlled colonies enable experimental infections and detailed characterization of virus–mosquito interactions and vector competence under reproducible conditions, informing risk assessment and outbreak preparedness (10, 11, 25–27). Colonies have also supported major progress in understanding insecticide resistance mechanisms, associated fitness costs, and resistance management strategies that are critical for sustaining intervention effectiveness (28–31). In addition, colonies facilitate studies of behavioral ecology, including host-seeking and oviposition-site selection, and allow testing of environmental stimuli and sensory cues that can be exploited for surveillance and control (1, 32, 33). Finally, mosquito colonies are foundational for evaluating and operationalizing innovative control approaches, including Wolbachia-based strategies and sterile/incompatible insect techniques, where consistent colony quality and biosecurity determine scalability and performance (24, 34–37).

## 2 Mosquito morphology and bionomics

Taxonomically, mosquitoes belong to the class *Insecta* within the order *Diptera* and the family *Culicidae*. This family is further classified into three subfamilies: *Toxorhynchitinae*, *Anophelinae*, and *Culicinae* (38, 39). Morphologically, mosquitoes are small insects measuring approximately 3–4 mm in length, characterized by their slender, delicate bodies and long legs. The mosquito’s body is divided into three main parts: the head, thorax, and abdomen, all covered with scales and setae of varying lengths and colors. These structures often serve as critical taxonomic markers for identifying mosquito genera and species (40, 41).

The head contains sensory organs, including compound eyes, segmented antennae, palps, and the proboscis, which morphologically vary between males and females. Female mosquitoes possess piercing and sucking mouthparts adapted for blood-feeding, with the proboscis containing components like mandibles and maxillae, enabling the penetration of host skin. Males, in contrast, lack these adaptations and do not feed on blood (42–44).

The thorax comprises three segments: prothorax, mesothorax, and metathorax, which support locomotion and respiration. This region is covered with scales that may vary in color and pattern among species, providing another taxonomic feature. A single pair of wings is affixed to the mesothorax, giving the mosquito its ability to fly. The second pair of wings is reduced to halteres, a defining feature of Diptera. Halteres function as gyroscopic organs that detect body rotations through mechanosensory feedback and provide rapid stabilization during flight, thereby enabling precise maneuvering and control. The wings are distinctive due to the presence of a full fringe of large scales along the wing margin and each vein, contributing to their unique appearance and aiding in species identification (45, 46). Additionally, each thoracic segment is associated with a pair of legs, contributing to the mosquito’s agility. The legs are long, slender, and delicate, providing agility and mobility (45–47).

The thorax, therefore, not only facilitates locomotion and respiration but also plays a crucial role in the mosquito’s ability to navigate and adapt to its environment effectively (41, 47–49). The mosquito abdomen, which consists of ten segments, plays key roles in respiration and reproduction. Females require blood meal, rich in proteins for egg development. This behavior highlights their ecological importance in disease transmission. The abdomen can distend significantly after feeding, accommodating developing eggs (42, 50–52).

### 2.1 Internal physiological systems relevant to colony maintenance

Colony performance is also shaped by internal physiology. The respiratory system, based on a tracheal network and spiracles, is sensitive to microclimate and crowding; inadequate ventilation and excessive organic load in larval trays can reduce oxygen availability and increase stress during development (1, 48). The digestive system links husbandry directly to fitness: larvae depend on microbial/particulate food in water, while adults rely on sugar for energy and females require blood to initiate vitellogenesis and egg maturation, making diet quality and feeding frequency key determinants of fecundity (51, 53, 54). The nervous system and sensory pathways regulate host-seeking, mating, and oviposition behavior; therefore, photoperiod, cage design, and environmental cues can influence feeding success and egg laying under insectary conditions (45, 55). Finally, the reproductive system is tightly coupled to nutritional and hormonal regulation; variation in temperature, blood-feeding success, and blood source can alter gonotrophic cycle length and egg output, affecting generational turnover and colony stability (1, 56, 57).

### 2.2 *Anopheles* species

**Figure 1:**
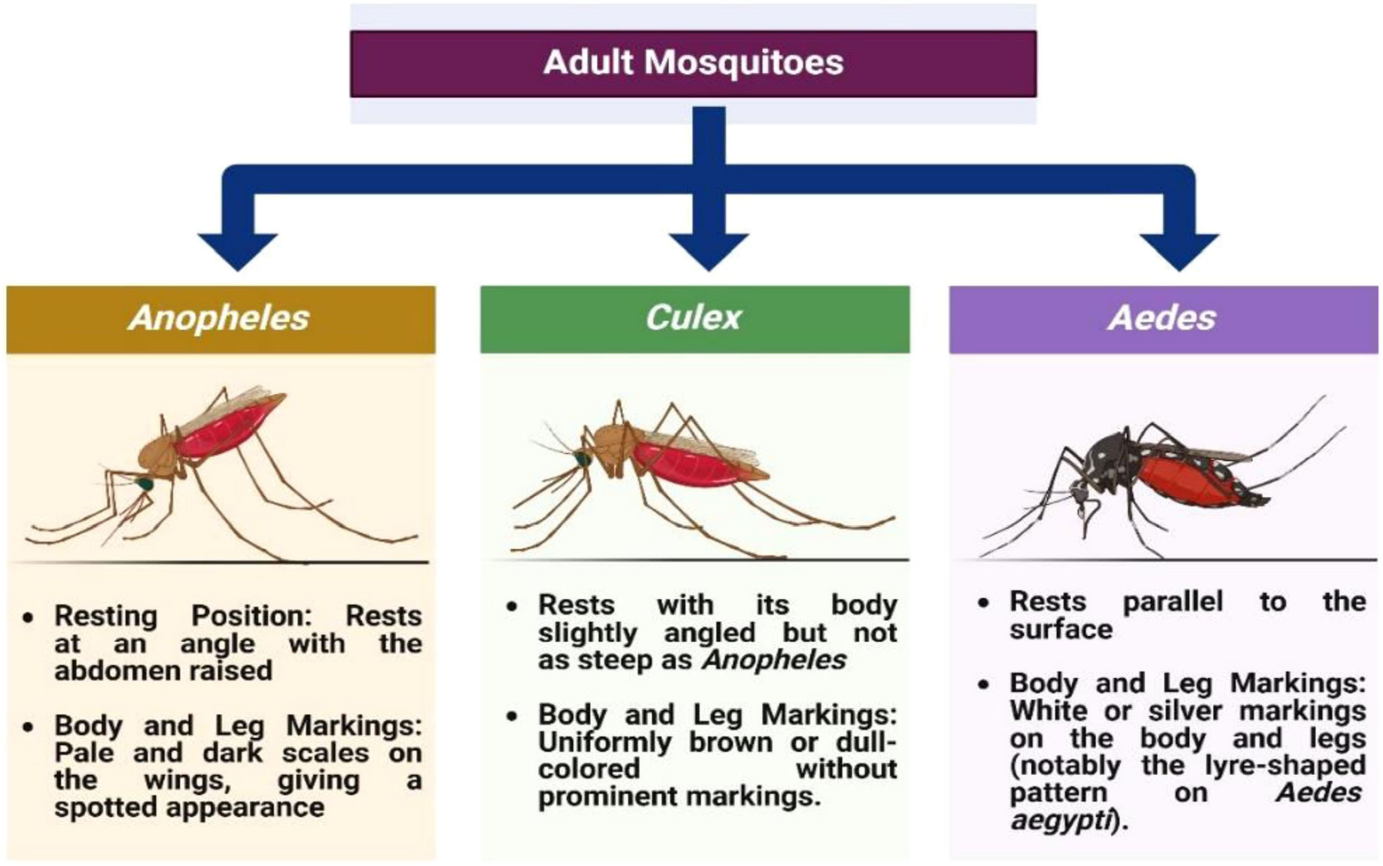
Differentiation of eggs at the genus level (*Anopheles*, *Culex*, and *Aedes*). Source: Created in BioRender. Pune 1, N [Internet]. 2025. Available from: https://BioRender.com/.

*Anopheles* mosquitoes are the primary vectors of human malaria and remain central to malaria epidemiology and control (1, 6, 7, 58). Adult *Anopheles* are typically dark brown to black and show distinctive morphological traits. Females are often recognized by their characteristic resting posture, in which the proboscis and abdomen align in a near-straight line (**Figure 1**). Unlike culicine mosquitoes, *Anopheles* generally lack appressed scales on the dorsal and ventral abdominal surfaces. In both sexes, the palps are as long as the proboscis, and in males the palps are characteristically clubbed at the tips (45, 59). The wings bear dark and pale scale patches arranged in species-specific patterns that support taxonomic differentiation (59–61).

Globally, malaria transmission is mediated by a small number of highly efficient *Anopheles* vectors, and the dominant species vary by region. In sub-Saharan Africa, key vectors include the *Anopheles gambiae* complex (notably *An. gambiae* and *An. arabiensis*) and the *An. funestus* group. In South and Southeast Asia, major vectors include *An. stephensi* (notably an important urban vector), *An. culicifacies* and *An. fluviatilis* (a principal rural vector in the Indian subcontinent), *An. minimus* and *An. dirus* (important vectors in forested and foothill ecologies across parts of Southeast Asia). In the Americas, malaria transmission is strongly associated with *An. darlingi* in many endemic settings. These species/complexes differ in larval habitat preference, biting/resting behavior, and seasonality, which directly affects local vector control strategies and the feasibility of colonization and maintenance under insectary conditions (1, 7, 12, 58).

**Figure 2:**
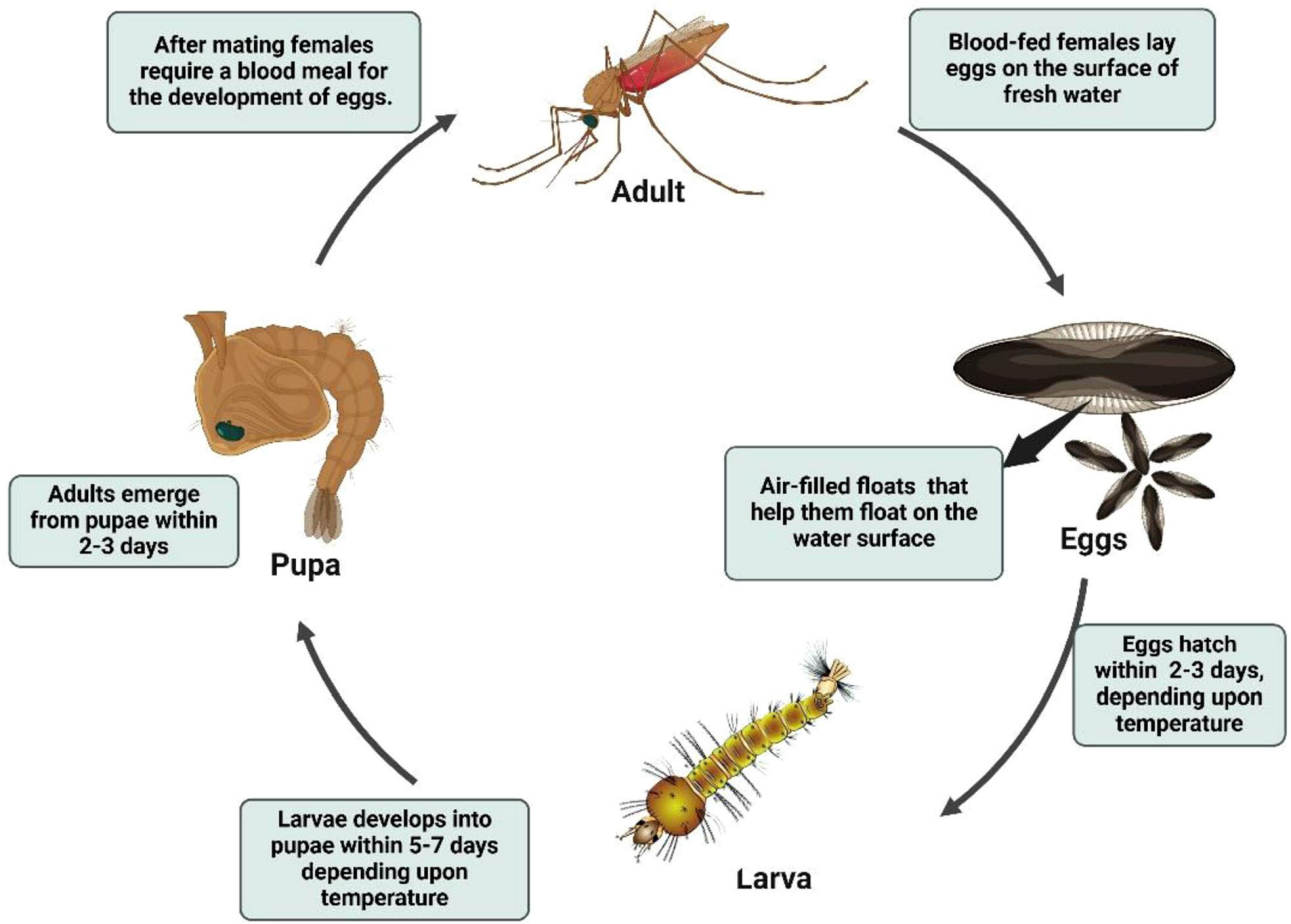
Life cycle of *Anopheles* mosquito. Source: Created in BioRender. Pune 1, N [Internet]. 2025. Available from: https://BioRender.com/.

**Figure 3:**
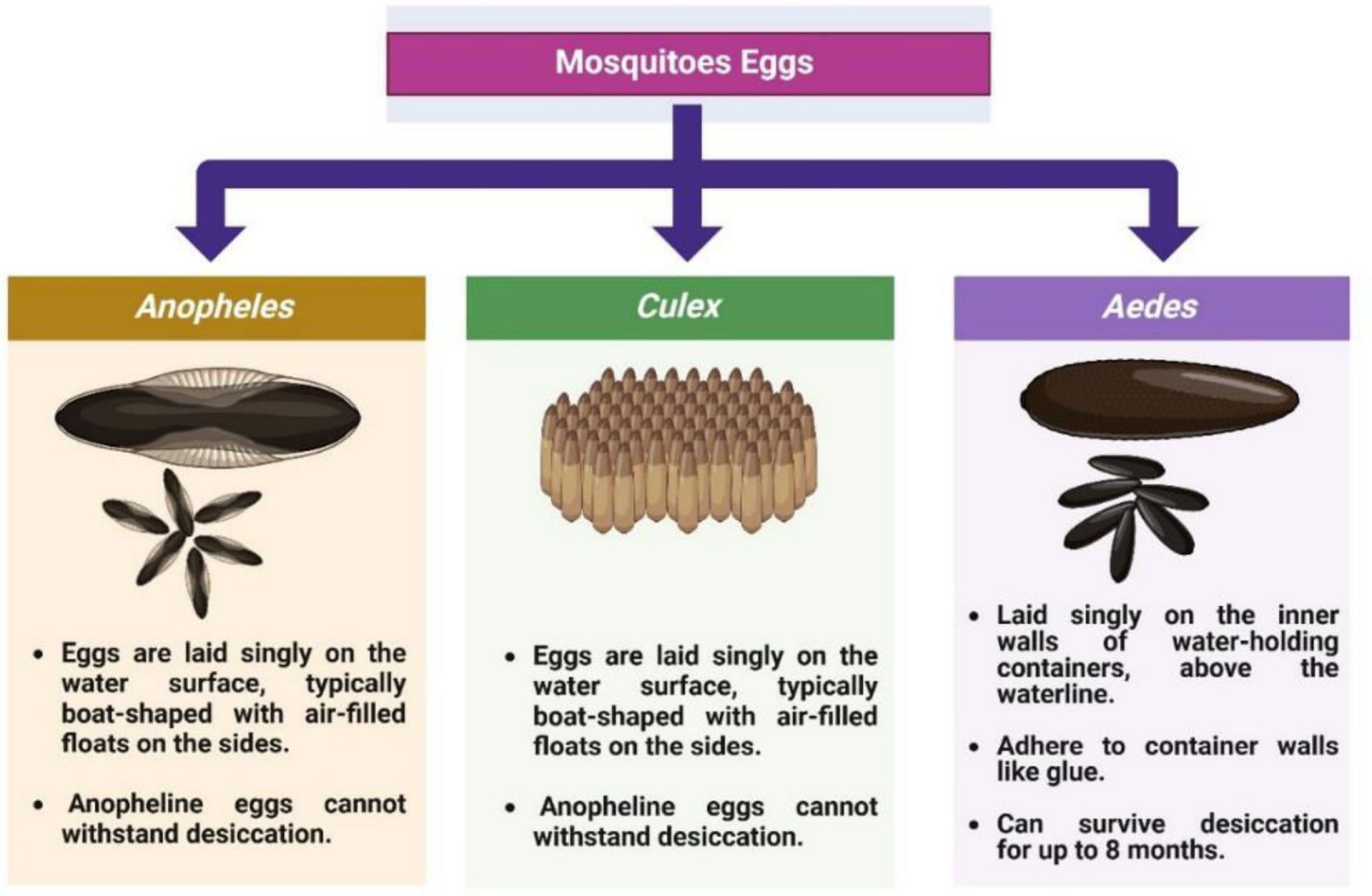
Differentiation of eggs at the genus level (*Anopheles*, *Culex*, and *Aedes*). Source: Created in BioRender. Pune 1, N [Internet]. 2025. Available from: https://BioRender.com/.

**Figure 4:**
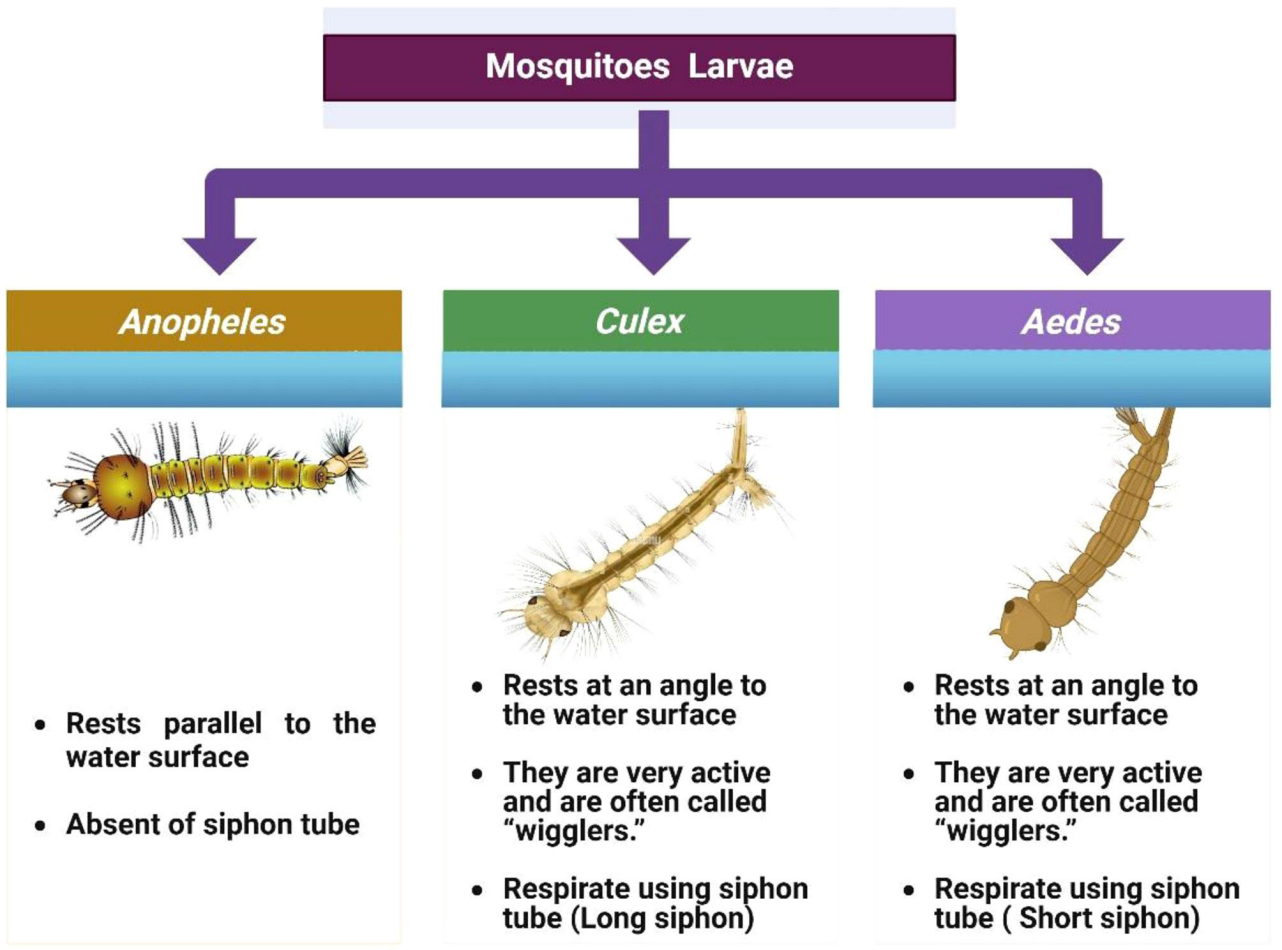
Differentiation of larvae at the genus level (*Anopheles*, *Culex*, and *Aedes*). Source: Created in BioRender. Pune 1, N [Internet]. 2025. Available from: https://BioRender.com/.

The *Anopheles* life cycle comprises four stages egg, larva, pupa, and adult with aquatic development typically lasting 7–14 days, depending on temperature and nutritional availability (12, 62, 63) (**Figure 2**). Eggs are boat-shaped and possess lateral floats that maintain buoyancy at the water surface (**Figure 3**) (1, 58). Larvae hatch within 2–3 days, pass through four instars, and characteristically lack a siphon, resting parallel to the water surface while feeding on microorganisms and organic debris (**Figure 4**) (1, 12, 38, 58, 61). Pupae are active, non-feeding aquatic forms with relatively short, broad respiratory trumpets, and adults emerge 1–2 days after pupation (38, 45, 64).

Newly emerged adults rest briefly on the split pupal exuviae until the wings harden and flight is possible. Females are hematophagous and typically seek blood meals to support egg development; biting activity is most often nocturnal, although patterns vary by species and setting (1, 58). Oviposition commonly occurs in a wide range of aquatic habitats, including clean freshwater bodies such as wells, rain pools, tanks, and other site types depending on the local vector species (1, 12, 58). These bionomic traits directly shape malaria transmission and underpin vector control strategies (12, 64–68). Accordingly, understanding their life cycle, feeding behavior, and breeding ecology remains essential for effective malaria control and transmission reduction (1, 58, 69).

*Anopheles* mosquitoes exhibit marked ecological heterogeneity, which strongly influences malaria risk. Larval habitats include sunlit pools, seepages, stream margins, rice-field fringes, and a variety of man-made sites (e.g., wells, borrow pits, construction-related water bodies), with habitat preference varying across species complexes and landscapes (1, 58, 69). Adult behavior is similarly diverse: vectors may be predominantly endophagic/endophilic (feeding and resting indoors) or exophagic/exophilic (outdoor biting and resting), affecting the impact of interventions such as indoor residual spraying and insecticide-treated nets (1, 58). Peak biting often occurs from dusk to dawn, but biting time, host preference (anthropophily *vs*. zoophily), and seasonal abundance can shift with ecology, host availability, and control pressure key determinants of vectorial capacity and local transmission intensity (64). In laboratory colonization, these ecological and behavioral differences can influence mating success, blood-feeding acceptance, and oviposition, highlighting the need for species-specific insectary conditions and routine monitoring of behavioral traits across generations (1, 45, 58).

### 2.3 *Culex* species

*Culex* is a widespread mosquito genus found across much of the world, except in extreme northern and southern temperate regions. These mosquitoes belong to the subfamily Culicinae and are often referred to as “culicine mosquitoes (70, 71).” They are particularly associated with human habitations and agricultural settings, such as rice fields. Notable members of the *Culex vishnui* subgroup include *Culex tritaeniorhynchus*, *Cx. pseudovishnui*, and *Cx. vishnui* (12, 46, 72).

The external morphology of *Culex* mosquitoes is characterized by pale, often brown colored, scales covering the thorax, legs, and wing veins, with brown or blackish scales on most abdominal segments. Females have rounded abdominal tips, and their palps are much shorter than the proboscis, whereas males possess palps nearly as long as their proboscis. Eggs are laid in rafts containing up to 300 eggs bound together in a boat-shaped structure (**Figure 3**). These cylindrical, brown eggs are kept afloat on water surfaces through surface tension and adhesion, supported by hydrophobic outer and hydrophilic inner surfaces (1, 12, 48, 61, 70).

Larvae of *Culex* mosquitoes have long, narrow siphons and lack abdominal palmate hair and tergal plates. They hang upside down at an angle from the water surface while breathing (**Figure 4**). These larvae progress through four developmental stages (instar I, II, III, IV), molting between each stage before pupation. The comma-shaped pupae are aquatic and highly active, with respiratory trumpets that are longer and cylindrical. Pupae do not feed and eventually split their cases to release adult mosquitoes, which rest with their thorax and abdomen parallel to surfaces (1, 73–75).

**Figure 5:**
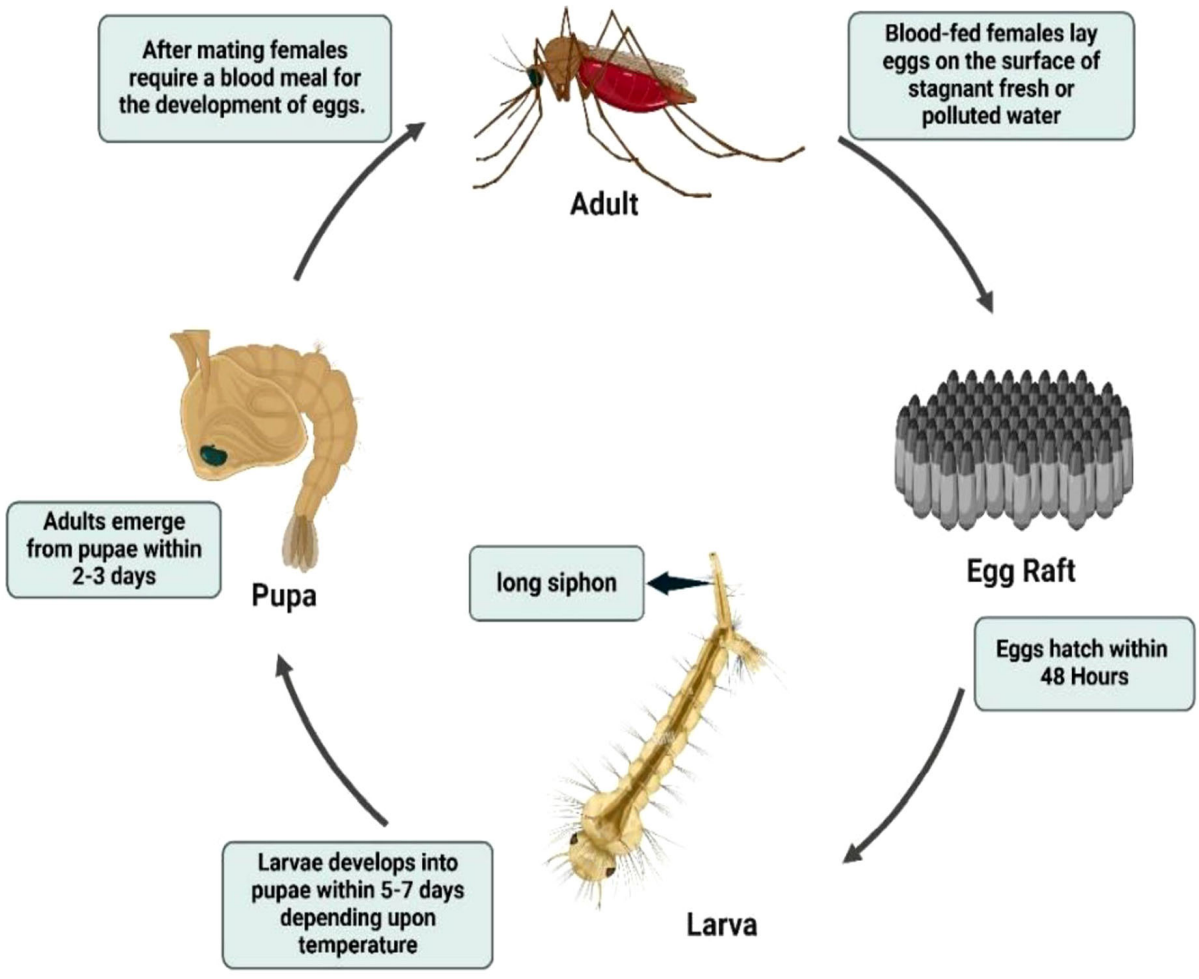
Life cycle of *Culex* mosquito. Source: Created in BioRender. Pune 1, N [Internet]. 2025. Available from: https://BioRender.com/.

The lifecycle of *Culex* mosquitoes from egg to adult spans approximately 10–14 days and is often favored by urbanization and poor drainage systems (**Figure 5**). Female *Culex* mosquitoes typically oviposit in stagnant or polluted water, including drains, septic tanks, marshy sites, shallow ditches, rice fields, and artificial containers (e.g., barrels and tins), where egg rafts hatch within 24–48 h into free-swimming larvae. Larvae pass through four instars and generally pupate within about one week, with adults emerging 1–2 days later under favorable conditions (12, 61, 71, 76). Adult females are hematophagous and require a blood meal for egg development, whereas males rely on plant sugars. Ecologically important species show habitat preferences relevant to disease transmission: *Cx. quinquefasciatus* (a major vector of lymphatic filariasis) commonly proliferates in organically rich, polluted urban water, whereas *Cx. tritaeniorhynchus* (a key Japanese encephalitis vector) is strongly associated with irrigated agricultural habitats, particularly rice fields (77). *Culex* mosquitoes usually disperse within a few kilometers (often ~2–3 km), and feeding behavior varies by species; for example, *Cx. quinquefasciatus* is highly anthropophilic and tends to bite indoors at night, while other *Culex* species may feed more readily on domestic animals (78, 79).

Although the broad range of *Culex* breeding habitats is well documented, an important remaining need is systematic mapping and quantification of the most productive larval sites across settings (e.g., urban drains and septic systems, peri-urban ditches, and irrigated agriculture), because larval presence does not always translate into high adult output. Such mapping can support targeted larval source management and improve prioritization of control resources (1, 12, 69). Adult feeding behavior is also comparatively well described but remains context-dependent, with many *Culex* vectors showing predominantly nocturnal biting and feeding occurring indoors or outdoors depending on local ecology. Host choice and biting location can vary with host availability, seasonality, urbanization, and vector control pressure, with direct implications for surveillance design and intervention impact (1, 45, 64).

Effective control of *Culex* mosquitoes therefore depends on identifying locally productive breeding sites and understanding where, when, and on whom mosquitoes feed, particularly in urban and peri-urban areas affected by inadequate sanitation and drainage. This ecological evidence base is essential for implementing targeted vector control strategies to reduce lymphatic filariasis and Japanese encephalitis risk. (12, 37, 64, 80).

### 2.4 *Aedes* species

*Aedes* is a widely distributed mosquito genus, originally confined to tropical and subtropical zones, but now present across all continents except Antarctica due to increased human movement and urbanization (45, 61, 80–83). The geographical range of *Aedes* species extends into northern and arctic regions. Among its members *Ae. aegypti* and *Ae. albopictus* are the most medically significant, serving as vectors for diseases such as Dengue, Chikungunya, Zika, and Yellow fever (84–86). Adult *Aedes* mosquitoes exhibit sexual dimorphism, with males being smaller and having plumose antennae, while females possess shorter palps and non-plumose antennae. The thorax is adorned with black, white, or silvery scales, forming conspicuous patterns, though some species may feature yellow or brownish scales. The wings are covered with narrow black scales, and the abdomen, consisting of eight segments, displays black and white scales in distinctive patterns. Females are characterized by pointed abdominal tips, which, in *Ae. aegypti*, are accompanied by the absence of spiracular bristles. Notable features include dark and white rings on the legs and lyre-shaped silver markings on the scutum of *Ae. aegypti*, contrasting with the median white stripe of *Ae. albopictus* (1, 48, 87–89).

**Figure 6:**
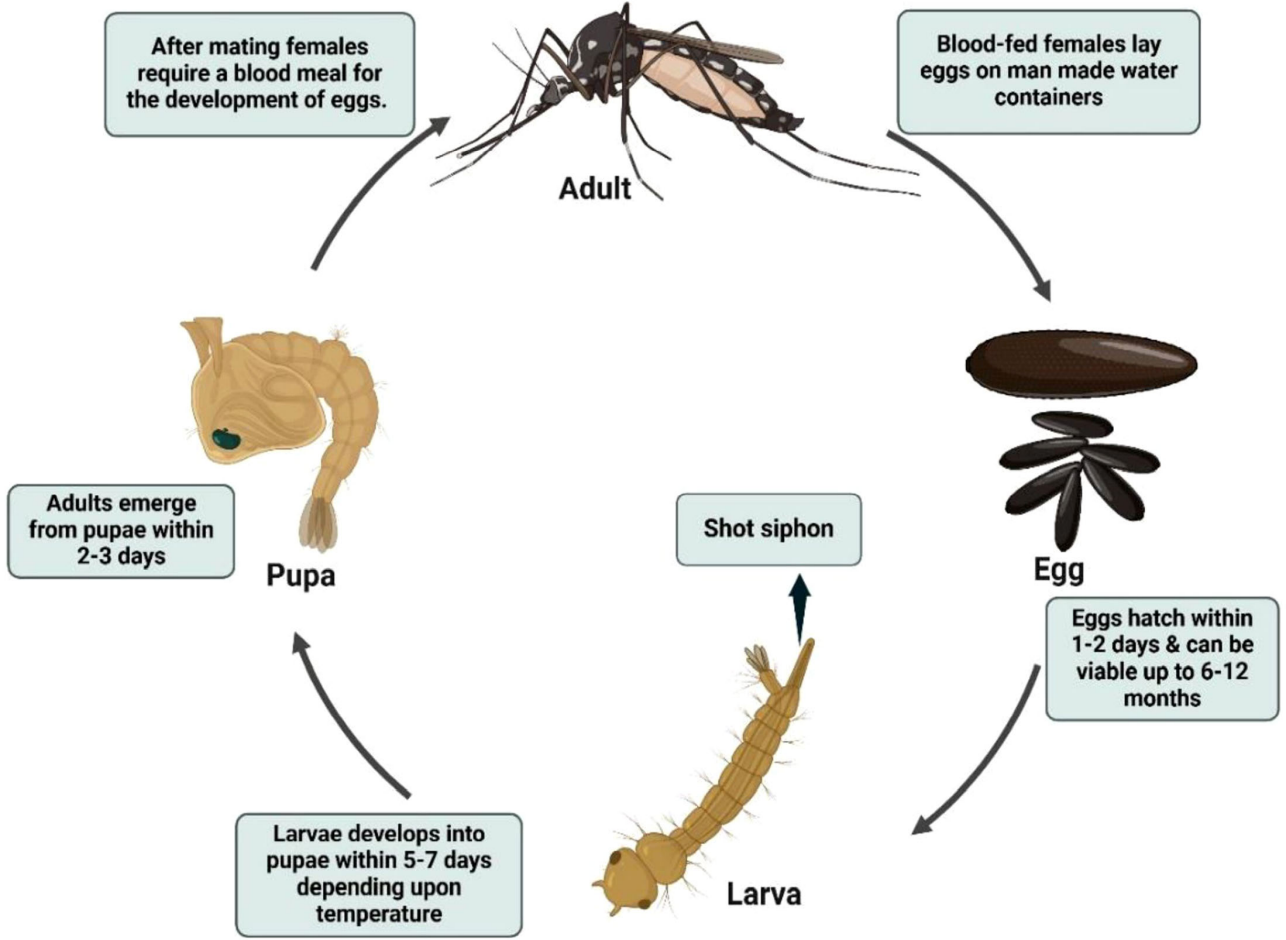
Life cycle of *Aedes* mosquito. Source: Created in BioRender. Pune 1, N [Internet]. 2025. Available from: https://BioRender.com/.

The lifecycle of *Aedes* mosquitoes spans approximately 10–12 days, heavily dependent on water availability and ambient temperature (**Figure 6**) (84). Females lay eggs on damp surfaces above the waterline, often in natural or artificial containers such as tree holes, tires, jars, and desert coolers (1, 87, 90, 91). Initially white, eggs turn black and can survive desiccation for months, hatching when submerged in water. The larvae feed on organic matter and algae in water and possess a short, barrel-shaped siphon for respiration. Larvae hang from the water surface at an angle and undergo four developmental instars before pupating (**Figure 3**). The pupal stage typically lasts 1 to 2 days, during which the mosquito does not feed but remains active, evading threats by tumbling through the water. Adult mosquitoes emerge from the pupal case at the water surface and become capable of flight once their wings have fully expanded and hardened (1, 12, 84, 88, 92, 93).

*Ae. aegypti* is a highly anthropophilic mosquito, commonly found in human-made environments, breeding in water containers and resting indoors in dark corners. It bites during the day and prefers urban or peri-urban settings. Its density peaks during monsoon and post-monsoon seasons and is linked to water storage practices in arid regions (12, 94–96; 97, 98). *Ae. albopictus*, originally from Southeast Asia, is a secondary vector, adapting to peri-domestic and natural habitats such as tree holes and vegetation. The spread of *Ae. albopictus* to new regions has been facilitated by the transport of egg-laden items like used tyres (84, 88, 99–101). The adaptability of *Aedes* mosquitoes to diverse environments and resilience in adverse conditions highlights the need for targeted control strategies, such as habitat management and public awareness initiatives, to reduce their impact on public health (89, 102–106).

## 3 Optimal environmental conditions for development of mosquitoes

Mosquito development, survival, and reproduction are shaped by interacting abiotic and biotic factors, including temperature, relative humidity, water quality, habitat type, and nutritional resources. Together these variables influence mosquito physiology and behavior, ultimately determining population dynamics and vectorial capacity (106, 107).

Temperature is a primary determinant of immature development rate and adult fitness. In general, increasing temperature accelerates larval development and shortens generation time, but extremes can increase mortality and produce smaller adults with reduced longevity and altered reproductive performance (106, 108). For example, in *Culex* mosquitoes, development increases with temperature across commonly tested ranges and is often optimal around 25-30 °C in laboratory settings (109). Temperature responses may differ between field and colonized populations, highlighting the importance of context-specific validation when translating laboratory findings to natural systems (109). Temperature-dependent effects are also evident in *Anopheles*, where thermal conditions influence the gonotrophic cycle, survival, biting frequency, and longevity; species may vary in heat tolerance, with some vectors showing greater resilience under higher temperatures than others (60, 110–112). In *Aedes*, temperature affects egg development and hatch success, larval survival, development time, and adult fecundity; extreme temperatures can increase immature mortality and may shift population traits important for transmission (106, 113, 114).

Relative humidity strongly modulates adult survival, activity, and reproductive success, and its effects can interact with temperature. In *Anopheles*, survival is generally favored by moderate-to-high humidity, whereas very low humidity imposes strong desiccation stress and sharply reduces lifespan (115–117). Humidity is also especially relevant for anthropophilic culicines (*Aedes* and *Culex)* maintained in insectaries. In *Aedes*, low humidity reduces longevity and increases desiccation stress, while moderate-to-high humidity supports higher survival and oviposition performance under standard insectary temperatures (1, 58, 106, 118). *Aedes* eggs are laid on damp substrates above the waterline and typically require a short humid embryonation period before they are dried for storage. Excessively low humidity during embryonation or prolonged storage can reduce egg viability and hatch success (1, 58). In *Culex*, adult activity and survival similarly improve under moderate humidity, whereas dry conditions may reduce longevity and compromise blood-feeding success and fecundity, thereby lowering colony productivity (1, 45, 69). These observations support insectary practices that maintain relatively stable humidity (commonly ~70–80% RH) to minimize physiological stress and improve colony consistency (1, 36, 58).

Beyond temperature and humidity, water quality influences larval nutrition and survival by shaping microbial and algal resources within breeding containers. However, “poor water quality” is species dependent: *Ae. aegypti* commonly thrives in relatively clean container water, whereas *Cx. quinquefasciatus* can proliferate in organically rich, polluted habitats; excessively contaminated or nutrient-depleted water can reduce immature survival and adult emergence (17, 21, 32).

Nutrition also affects key life-history traits relevant to laboratory rearing and vector competence. Larvae feed on microorganisms and detritus in aquatic habitats, and larval nutritional status shapes adult longevity, flight capacity, and competence for pathogen transmission (119). Adults rely primarily on sugar sources for energy, while females additionally blood-feed to obtain nutrients required for oogenesis; blood-meal quantity and quality influence egg production, and micronutrients such as iron contribute to successful egg development (51, 53, 57, 120). Laboratory rearing protocols therefore aim to standardize larval diets and adult feeding to maintain consistent mosquito size and performance across generations (121).

Finally, environmental cycles of temperature, humidity, and photoperiod modulate circadian rhythms that influence mating, feeding, and oviposition behaviors, which can affect colony productivity and experimental outcomes (32, 55). Collectively, these environmental determinants also affect pathogen development within mosquitoes, influencing transmission potential and the likely impacts of climate variability and change (13, 107, 122, 123). Optimal temperature and humidity ranges for key mosquito species are summarized in **Table 1**.

**Table 1:** Optimal temperature and humidity for growth, development, and survival of different mosquito species.

Sr. no.	Genus	Species	Optimal temperature	Optimal humidity	References
1.	*Aedes*	*Ae. aegypti*	26 °C - 28 °C	75%-80%	(118)
		*Ae. albopictus*	25 °C - 28 °C	75%-80%	(124)
		*Ae. vittatus*	24 °C - 27 °C	75%-80%	(125)
2.	*Culex*	*Cx. quinquefasciatus*	26 °C - 28 °C	70%-80%	(109)
		*cx. tritaeniorhynchus*	26 °C - 30 °C	55%-60%	(126)
		*cx. gelidus*	27 °C - 28 °C	80%	(127)
3.	*Anopheles*	*An. stephensi*	26 °C - 29 °C	70%-80%	(128)
		*An. culicifacies*	28 °C - 30 °C	60%	(129)
		*An. minimus*	24 - 26 °C	70%-80%	(130)
		*An. dirus*	23 - 25 °C	70%-75%	(131)
		*An. fluviatilis*	27 °C - 29 °C	80%-85%	(132)
		*An. philippinensis*	25 °C - 27 °C	70%-80%	(133)

## 4 Mass rearing and colonization of mosquitoes

Establishing a mosquito colony in a laboratory requires insectary equipment, meticulous preparation, specialized tools, and close adherence to biosafety regulations. In the context of this review, “mass rearing” refers to the maintenance of mosquito colonies at a scale sufficient to support experimental, operational, and training needs. Specifically, we define a mass rearing batch as a unit involving at least 1,000-2,000 larvae per species per cycle, maintained in multiple trays under controlled environmental conditions. For adult colonies, this corresponds to rearing 2,000-5,000 mosquitoes within a 1-2-week period, depending on species and experimental requirements. These numbers are consistent with mass production standards reported in sterile insect technique (SIT) and vector competence studies. The field collection of eggs, larvae, or adult mosquitoes represents the initial step in establishing a mosquito colony. Depending on the study’s objectives, team members may collect eggs, larvae, or adults, as well as various localized subspecies (21, 24, 134).

Colony maintenance models. Laboratory mosquito colonies are typically maintained under two broad operational models: (i) research colonies (small to medium scale) optimized for experimental reproducibility (e.g., vector competence, physiology, microbiome studies), and (ii) mass-rearing colonies designed to generate large cohorts for operational and translational applications (e.g., SIT/IIT, large-scale phenotyping, product evaluation). These models differ in cage design, larval density management, feeding logistics, quality control metrics, and biosecurity requirements (17, 20, 22, 24, 36, 135, 136).

### 4.1 Importance of colony adaptation to laboratory conditions

For a mosquito colony to be successfully established, it must be well adapted to laboratory conditions. Humidity and temperature play a crucial role in mosquito rearing, with optimal settings at 27 °C ± 2 and relative humidity at 75% ± 10 (17, 24). The simplest method to regulate humidity and heat involves using a wet towel draped over the cage and a small electric light bulb (20, 137). More advanced systems, such as environmental chambers with programmed electronic controls for temperature, humidity, and photoperiod, provide precise regulation. The choice of a humidity and temperature control system depends on the size and requirements of the insectary (21). Optimal mosquito colony growth and development occur with a 14-hour light and 10-hour dark cycle (21, 138).

### 4.2 Standard operating procedures: rearing and colonization of mosquitoes

Rearing practices vary in complexity, from basic setups to sophisticated facilities equipped with environmental controls. Despite these differences, the ultimate goal is to ensure the availability of reliable and cost-effective mosquito colonies for research and training. (17, 22, 24, 139).

### 4.3 Materials and infrastructure

#### 4.3.1 Essential materials

**Figure 7:**
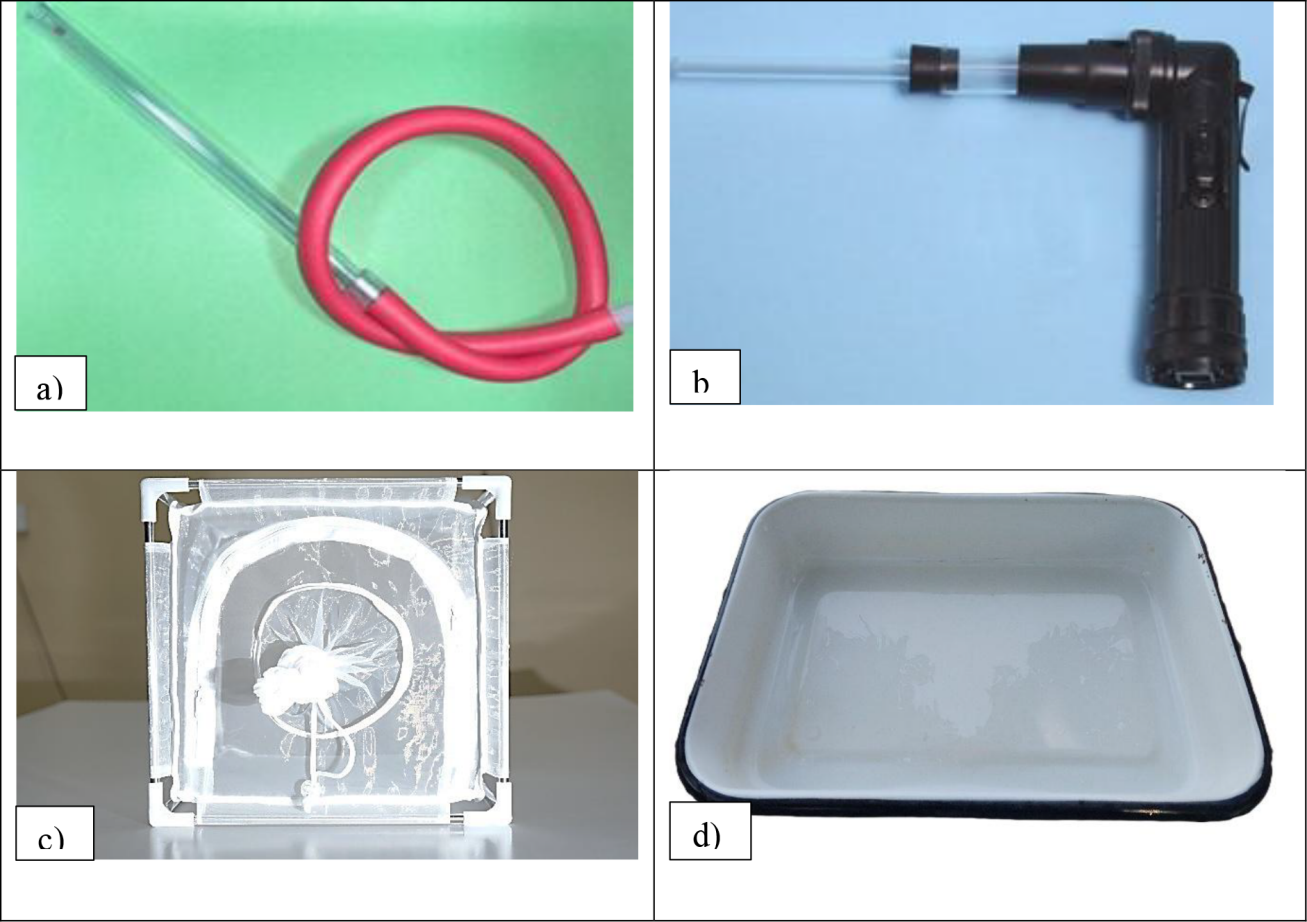
Materials for mosquito rearing and colonization: **(a)** oral aspirator, **(b)** mechanical aspirator, **(c)** mosquito cage, **(d)** larval tray.

Rearing mosquitoes requires specialized equipment and supplies designed for the various developmental stages of mosquitoes. The following materials are essential for a mosquito-rearing facility:

- Aspirators: For collection and transferring mosquitoes (**Figures 7a, b**).
- Cages: Fiber-framed, 30×30×30 cm, with double mosquito netting or commerciallyavailable Bugdorm cages (1 cubic foot) to house approximately 200–500 adults (**Figure 7c**).
- Cage categories: Adult holding systems range from small research cages (e.g., 30×30×30 cm fabric cages or commercial cages) suitable for routine colony maintenance, to mass-rearing cages engineered for high adult density, efficient blood feeding, and simplified egg collection. Mass-production cage designs have been developed and evaluated for *Aedes* and are commonly used in SIT/IIT-aligned workflows, where cage architecture affects mating success, feeding rates, egg yield, and labor requirements (20, 24, 136, 137). Cage choice should match colony scale, biosafety level, workflow (infection *vs* routine), and available environmental control capacity (36).
- Rearing Containers: Enamel trays (3–5 liters capacity) for larvae and enamel bowls (300 ml capacity) for oviposition.
- Feeding Equipment: Larval food: A 60:40 mix of dog biscuits and yeast. Adult feeding materials: 10% glucose solution and adult feeding apparatus and chicken, bovine, or human blood.
- Environmental Controls: Air conditioners and humidifiers fitted with humidistats to maintain optimal temperature and humidity.
- Miscellaneous Tools/things: Stereomicroscopes for adult identification, parafilm membranes for blood feeding, thermometers, and data loggers for environmental monitoring, electric mosquito zapper/racket.
- Basic PPE requirements during mosquito rearing include: Lab coats or aprons, Gloves (latex or nitrile), Face masks (especially during blood feeding or cleaning) and Closed footwear.

#### 4.3.2 Environmental conditions

Rearing mosquitoes requires maintaining specific environmental conditions for optimal development and survival: Temperature: 27 °C ± 2; Humidity: 75% ± 10; Water Source: Dechlorinated Tap water, tested biannually for quality. Regular monitoring and adjustments ensure consistency and minimize disruptions to the colony.

#### 4.3.3 Rearing procedures

Core steps in colony establishment and routine maintenance. Colony establishment typically begins with field-collected eggs/larvae/adults, followed by (i) species confirmation (morphological and/or molecular), (ii) founder expansion while minimizing bottlenecks, (iii) stabilization of feeding/oviposition in insectary conditions, and (iv) routine cyclic maintenance (egg collection, larval rearing, pupal harvest, adult emergence, sugar feeding, blood feeding, oviposition) with standardized schedules and record keeping. Routine maintenance is strengthened by periodic checks of key performance indicators such as larval development time, pupation rate, adult emergence, blood-feeding rate, fecundity/egg hatch, and adult longevity, which are recommended in established colony maintenance manuals and mass-rearing SOPs (22, 24, 36, 135, 136).

#### 4.3.4 Larval food preparation

- Ingredients: Mix dog biscuits and yeast in a 60:40 ratio. Dog biscuit–yeast diets are widely used for routine mosquito colony maintenance because they are inexpensive, locally available, and generally support acceptable development and emergence when larval density and feeding schedules are well controlled (21, 22, 24, 135). However, diet choice should be guided by the purpose of rearing and quality targets. Protein-rich diets can accelerate development and increase pupal output but may also increase water fouling and microbial overgrowth if overfed, which can elevate larval mortality and produce uneven adult size. Conversely, more balanced or standardized formulations may improve survivorship and adult uniformity, which is important for reproducible vector competence and intervention studies. Comparative assessments in *Aedes* mass rearing demonstrate that diet formulation can affect development time, emergence, adult size, and colony productivity, highlighting trade-offs between rapid production and adult quality (137, 140–142). Therefore, we selected this commonly used methodology as a practical baseline but emphasize that laboratories should optimize and report diet composition, feeding frequency, larval density, and water management to match their experimental objectives (24, 119).
- Preparation: Grind the mixture into a fine powder using a mixer grinder. Sieve to remove coarse particles.
- Sterilization (if required): Autoclaving is optional and may be performed in studies requiring sterile larval conditions. For routine rearing, freshly prepared and properly stored food is sufficient.
- Storage: Store the prepared larval food in a clean, labelled, air-tight container at room temperature for up to one month.

#### 4.3.5 Larval rearing

a. Setup: Fill enamel trays (45×30×5 cm) with ~ 3 litters tap water.

b. Density Management: Introduce 500 larvae per tray to avoid overcrowding.

Note: For density control, it is recommended to introduce larvae from a pre-counted batch of eggs (e.g., 500–600 eggs per tray), ensuring uniform larval numbers after hatching.

c. Labelling: Use color-coded stickers to differentiate species: depending on laboratory follow-ups.

#### 4.3.6 Larval feeding

a. First and Second Instar: Provide 5 mL of diluted larval food (0.5 mg/larva) per tray. To prepare the food solution, mix 250 mg of larval food in 5 mL of water to ensure adequate nutrition for the larvae. For optimal larval development, the water in the container should be changed after the first and second instar stages, just before the larvae progress to the third and fourth instars. This helps remove accumulated waste and debris from previous feedings, maintaining a clean environment for further growth.

b. Third and Fourth Instar: Increase the food to 500 mg per tray (1 mg/larva).

Daily feeding is essential, with gentle stirring of trays to distribute food evenly. Throughout the larval feeding process, trays should be covered with a plastic lid fitted with a net to prevent escaped adults from laying eggs in the larval tray. This measure helps prevent cross-colony contamination.

c. Alternative larval diets: Although dog biscuit–yeast mixtures are widely used for routine colony maintenance, multiple alternative diets have been reported, including formulations based on fish food flakes/pellets, liver powder/yeast blends, cereal-based diets, and standardized mass-rearing diets optimized for growth rate and adult fitness. Comparative studies in *Aedes* have shown that larval diet composition influences development time, survivorship, adult size, and downstream traits relevant to vector competence and mass production outcomes (119, 137, 140–142). Novel approaches, such as supplementing diets using alternative insect-derived resources, have also been evaluated for cost-effective laboratory rearing (121).

#### 4.3.7 Adult feeding

a. Glucose Solution: Prepare a 10% glucose solution (50 g glucose in 500 ml water) and provide soaked cotton pads as a feeding source. Replace pads every two days.

b. Blood Feeding:

➢ Collect chicken or bovine blood in a sterile flask. To prevent clotting, add 1 mg heparin per 100 ml, or use glass beads as an alternative, particularly in virus-vector interaction studies where anticoagulants may affect results.
➢ Besides chicken and bovine blood, rabbit and guinea pig are also commonly used as blood-meal sources in some insectaries (either via direct feeding on restrained animals or by collecting blood for membrane feeding), depending on local ethics approvals, availability, and the mosquito species/strain. Host choice can influence feeding success, fecundity, and colony performance; therefore, the blood source should be standardized within a study and reported explicitly (1, 36, 58, 135).
➢ Warm the blood to 36.5–37.5 °C and offer it using a parafilm-stretched feeder.
➢ Allow adult mosquitoes (5 days old) to feed for 3–4 hours.

c. Membranes and artificial feeding systems. In addition to parafilm, mosquito colonies are maintained using a variety of membrane materials (including synthetic membranes) and artificial blood-feeding devices that regulate temperature and enable standardized feeding across cages. Multi-membrane or multi-reservoir systems have been described for *Aedes* and can improve throughput and reproducibility, particularly for mass-rearing or experimental infection workflows (24, 143–145). Because anticoagulants and blood handling can influence mosquito performance and some experimental outcomes, studies frequently standardize blood source, warming temperature, feeding duration, and post-feed handling to improve inter-batch consistency (24, 50, 51).

d. Anticoagulant selection and standardization. Blood is commonly collected with anticoagulants to prevent clotting, but the choice of anticoagulant may influence feeding success and, in some experimental contexts, biological readouts. Heparin is frequently used in routine colony maintenance, while alternative approaches include mechanical defibrination (e.g., glass beads) to minimize potential interference in pathogen vector studies where anticoagulants may affect downstream outcomes (24, 51, 145). Because different anticoagulants and handling conditions can alter blood quality and membrane-feeding performance, insectaries should standardize the blood source, anticoagulant (or defibrination method), warming temperature, and feeding duration within a study and report these parameters explicitly to ensure reproducibility (24, 50).

#### 4.3.8 Oviposition and egg collection

- Setup: Place ovitraps (enamel bowls lined with white filter paper) in adult cages 48 hours post-blood feeding. Fill the bowls to one-third with water.
- Species-Specific Protocols: *Anopheles*: Eggs are floated in trays for hatching; *Aedes*: Filter paper with eggs is dried for 48 hours and stored; *Culex*: Egg rafts are floated directly in larval trays.
- After oviposition, the eggs of *Aedes* mosquito are allowed to undergo embryonation by keeping the filter paper in a moist environment (80% relative humidity) for a period of 36–72 hours. These oviposition papers are then stored in lidded containers to allow for its gradual drying. For long-term storage, ideally up to 10 weeks, these dried oviposition papers can be kept in Ziploc bags under laboratory conditions. *Culex* and *Anopheles* continued hatching the eggs and maintaining the cycle.

#### 4.3.9 Pupae handling

- Separation: Pupae, which are darker and comma-shaped, can be visually distinguished and gently separated from larvae using a plastic pipette or dropper.
- Harvesting: Transfer 300–500 pupae to water-filled bowls within adult cages. Allow adults to emerge within two days.

#### 4.3.10 Facility maintenance

- Cleaning and Sterilization: Clean trays and cages with detergent, rinse thoroughly, and air-dry. Autoclave blood-feeding apparatus and other tools.
- Waste Disposal: Biological Waste- Treat dead mosquitoes and larvae with boiling water before disposal. Hazardous Waste: Disinfect blood waste with 1% sodium hypochlorite for one hour before disposal. General Waste: Separate biodegradable materials (e.g., cotton) for proper disposal.

#### 4.3.11 Data recording and monitoring

Record temperature and humidity at least three times daily during weekdays and twice on holidays. Maintain an entry/exit log for restricted access to the insectary.

#### 4.3.12 Colony purity and strain integrity

When insectaries maintain multiple mosquito species and/or parallel populations (e.g., insecticide-susceptible and resistant strains), routine verification of colony purity and strain integrity is critical to avoid inadvertent cross-contamination and misinterpretation of experimental results. Practical measures include strict physical separation of colonies (separate rooms or clearly zoned work areas), unambiguous cage/tray labeling, species strain-specific color coding, dedicated aspirators/forceps and feeding accessories for each colony, and handling workflows that move from ‘clean’ colonies to ‘high-risk’ colonies (e.g., resistant or infected lines) to minimize carryover. Routine quality checks should include periodic morphological confirmation of adults/larvae using diagnostic characters and, where feasible, molecular verification (e.g., COI barcoding or species-diagnostic PCRs) at defined intervals or after any suspected mix-up. These quality assurance steps align with widely used mosquito rearing manuals, SOP-based colony maintenance guidance, and arthropod containment/biosecurity recommendations (22, 24, 36, 135, 146).

#### 4.3.13 Generation management and disposal of older cohorts

To minimize overlapping generations and maintain reproducible colony age structure, colonies should be maintained in discrete cohorts with a defined schedule (e.g., synchronized egg collection, larval trays labeled by date, pupal transfer, adult emergence). Egg collections should be restricted to a planned window (e.g., 24–48 h after oviposition) and all trays/cages should carry the generation/date code. After the next generation is established (i.e., once sufficient eggs/larvae or pupae have been obtained), older adults should be removed from cages and residual immatures (remaining larvae/pupae) should be discarded to prevent carryover. This can be done by chilling/freezing adults prior to disposal and treating larval water with hot water or an appropriate disinfectant before discarding, following institutional biosafety and arthropod containment guidance. Cohort-based generation management reduces mixing of ages, improves consistency for experiments (e.g., standardized 3–7-day-old females), and helps limit unintended selection during long-term colonization (22, 24, 36, 135, 146).

#### 4.3.14 Maintaining insecticide-resistant strains and preventing reversion to susceptibility

Maintaining laboratory strains with defined phenotypes particularly insecticide resistance requires active quality control because resistance levels may decline over generations if insecticide exposure is removed. This can occur due to fitness costs associated with resistance alleles and relaxation of selection pressure, leading to partial or full recovery of susceptibility and reduced comparability across experiments. To address this, insectaries maintaining resistant strains should implement a documented strain-management plan that includes: (i) periodic phenotypic verification using standardized susceptibility bioassays at defined intervals (e.g., every 5–10 generations), (ii) where appropriate and ethically permitted, controlled selection using diagnostic concentrations or exposure regimes to maintain the resistance phenotype, (iii) complementary molecular monitoring of resistance markers when available, and (iv) strict separation of resistant and susceptible lines with dedicated equipment to prevent accidental mixing. Reporting the generation number, selection history, and assay outcomes improves reproducibility and interpretation of resistance-related findings (28, 30, 31, 139, 147–149).

#### 4.3.15 Challenges in mosquito rearing and colonization

Rearing and colonizing mosquitoes in laboratory settings present several challenges that impact efficiency and sustainability. Maintaining optimal environmental conditions, such as temperature (27 °C ± 2) and relative humidity (75% ± 10), is critical but often disrupted by external factors like power outages or inadequate infrastructure. Operational issues, including cross-contamination, improper larval density, and labor-intensive manual processes like pupal sorting, further complicate scalability. However, simpler methods such as mesh or sieve-based filtration systems are available and can improve efficiency in large-scale operations. Resource constraints, such as the high cost of materials and the need for skilled personnel, add to the difficulties, while ethical and regulatory hurdles in blood sourcing and waste disposal demand strict compliance. Genetic drift within colonies also poses a long-term challenge, necessitating the periodic introduction of wild strains to maintain biological relevance. Limited feeding, sorting, and monitoring automation compounds these issues, making scalability and consistency difficult. Addressing these challenges through technological advancements, better infrastructure, and training experience is essential for ensuring reliable and healthy mosquito colonies for research and training.

##### 4.3.15.1 Historical challenges in colony establishment and advances enabling successful colonization

Establishing new mosquito colonies from field populations has historically been constrained by a recurring set of operational and biological barriers. Early failures often reflected founder effects and bottlenecks (low initial numbers, skewed sex ratios, or low insemination rates), which reduced effective population size and increased the risk of colony collapse or rapid laboratory adaptation. Additional constraints include species-specific mating behavior (e.g., stenogamy *vs* eurygamy), stress-induced reductions in blood-feeding acceptance, and difficulties in eliciting oviposition under captive conditions. At the larval stage, overcrowding, unstable water quality, and suboptimal diets can produce small, low-fitness adults with reduced fecundity, while hidden infections (e.g., microsporidia, fungi, or bacterial blooms) can cause abrupt mortality and loss of colonies. Importantly, these issues are not only operational: they can bias downstream experiments by altering adult size, longevity, biting propensity, microbiome composition, and susceptibility to infection, thereby affecting reproducibility and the external validity of findings derived from long-term laboratory strains (1, 36, 135).

Over the past two decades, several advances have improved establishment success and long-term stability. Key developments include (i) better environmental regulation (programmable temperature/humidity/photoperiod systems and continuous monitoring), (ii) cage designs that support mating and husbandry at scale (including mass-rearing cages and modular cage systems), (iii) standardization of larval density and diet with adjustments for species- and strain-specific growth trajectories, and (iv) improved blood-feeding approaches, including membrane-feeding devices and multi-feeder systems that increase feeding rates while reducing reliance on live animals where appropriate. In parallel, modern colony management increasingly emphasizes routine quality control, including species/strain purity checks (especially when multiple colonies are maintained), periodic assessment of key phenotypes (e.g., size, fecundity, survival), and early detection/containment of microbial contamination to prevent colony crashes (22, 36, 135).

A well-known example of a historically challenging vector is *Anopheles funestus*, a major malaria vector in sub-Saharan Africa. Compared with some *Anopheles* species that colonize more readily, *An. funestus* has often been difficult to establish and maintain due to stringent ecological requirements, sensitivity to insectary conditions, and challenges in achieving consistent mating, blood feeding, and oviposition. Recent progress in colonizing and maintaining *An. funestus* colonies has been linked to improved insectary control, refined feeding/oviposition routines, and careful management of larval rearing conditions and colony health. This experience illustrates why “standard” protocols must often be adapted and why documenting colonization challenges and solutions is essential for improving reproducibility and expanding the range of vector species available for experimental research (1, 36, 58).

### 4.4 Balancing quality and quantity in mass production

It is necessary to strike a balance between meeting the biological requirements of the species and attaining high production rates and economic efficiency while optimizing mass-rearing conditions for both juvenile stages (for pupal production) and adult egg production (20, 137). Adult cages must also maintain the mosquitoes’ behavior as closely as feasible to their natural state in order to fine-tune the rearing cycle. In addition to being affordable, the perfect cage would be manageable in terms of handling, upkeep, and space needs (137). The size of the cage must be carefully adjusted according to the number of mosquitoes it is intended to house to provide ideal circumstances for mating, feeding, and high survivor rates (17, 137). It is advised that furniture be constructed of rust-proof metal; plastic, or fiber glass. If possible, it should also have wheels to clean underneath and around the furniture regularly. Cages used for rearing are stored on shelves (24, 135). To prevent ant infestation, especially around 10% glucose sources, insect traps or barriers (such as oil-coated bowls or sticky pads) should be placed at the legs of the mosquito cages.

### 4.5 Maintaining colony health and reducing genetic drift

Laboratory colonies created from field-collected individual mosquitoes may not represent the original source population because of their susceptibility to genetic drift, selection, and/or bottleneck (150). Loss of genetic diversity also results in low adaptive potential and inbreeding depression (151). Additionally, the absence of selective pressures in laboratory settings may result in decreased later-life reproduction (151). Loss of rare alleles, declining heterozygosity and effective population size, and inbreeding can all impact post-colonization genetic diversity and associated biological traits. Additionally, changes in the mosquito’s normal microbial flora during colonization may influence immune responses and interactions with pathogens (150, 152–154). Genetic variations may intensify with the length of time since colonization. Therefore, before interpreting results from pathogen–vector model studies using colonized vectors, assessing the similarity between colony and field populations is crucial in determining whether founder effects or bottlenecks have occurred (150). Arias and colleagues discovered significant genetic divergence between laboratory and field populations of *An. albimanus* after 20 years in a laboratory colony, as indicated by the mitochondrial Cytb gene (FST = 0.37179) (150, 155). Similarly, Andrea gloria-soria and colleagues discovered in their study that the average allelic richness was calculated from *Ae. aegypti* laboratory colonies is less than that of wild collections across 12 multi-allelic microsatellite loci and laboratory colonies have decreased heterozygosity as compared to natural collections (5). In a small population of colonized mosquitoes, random mating can readily encourage inbreeding and genetic drift (156). Inbreeding and genetic drift must be minimized for mosquito colonies to remain healthy and genetically diverse. Here are some methods to achieve this:

- Maintaining large colony sizes can aid in the reduction of genetic drift as it provides a larger gene pool.
- Artificial selection methods can be used to preserve genetic variety by selecting for particular traits or attributes.
- Cryopreserving mosquito embryos or sperm can help preserve genetic material and reduce the loss of genetic diversity over time.
- Regular genetic monitoring of laboratory colonies helps detect changes in genetic diversity and enables timely corrective actions. Molecular markers, such as microsatellites and single nucleotide polymorphisms (SNPs), have been effectively used to monitor colony structure, inbreeding levels, and genetic drift in mosquitoes. For example, microsatellite analysis has been used to assess genetic variability in *Aedes aegypti* (157), while SNP genotyping has provided fine-scale resolution of population structure in both *Aedes* and *Anopheles* mosquitoes (118).
- Structured breeding programs, such as periodic introgression of field-collected individuals or rotational mating schemes, can help maintain genetic diversity and prevent inbreeding depression in laboratory colonies. For example, Powell & Tabachnick (158) described introgression strategies in *Aedes aegypti*, while Azrag et al. (159) demonstrated the effectiveness of rotational colony maintenance in *Anopheles arabiensis* used in sterile insect technique (SIT) programs. These approaches ensure colony health, preserve vector traits, and reduce the risk of laboratory adaptation (158, 160).
- It is also equally important to ensure that personnel involved in mosquito breeding are educated on the importance of genetic diversity and techniques to maintain it.

Cleanliness and sterility in the insectary should be maintained to prevent pests, and infestations of other potentially dangerous insects like book lice (Psocoptera), predatory ants, and roaches that can devastate egg stocks. Dust and scales shed by adult mosquitoes should be removed as frequently as possible because they can also cause severe respiratory complaints (24). Preventing bacterial and fungal infections is essential in maintaining a healthy and viable mosquito colony (22, 24, 137). The challenges in mosquito colony maintenance, along with their impacts and mitigation strategies, are summarized in **Table 2**.

**Table 2:** Challenges in Mosquito Colony Maintenance: Impacts and Mitigation Strategies.

Sl. No.	Challenges	Impacts	Mitigation Strategies
1	Genetic Drift and Bottlenecks	Loss of rare alleles, reduced effective population size	Maintain large colony sizes; structured breeding programs
2	Loss of Genetic Diversity	Low adaptive potential, inbreeding depression	Artificial selection; cryopreservation of embryos or sperm
3	Inbreeding and Reduced Heterozygosity	Decline in genetic variability and fitness	Regular genetic monitoring using molecular markers
4	Pathogen Interaction Impact	Altered biological responses to pathogens	Ensure genetic similarity between field and colony populations
5	Colonization Divergence Over Time	Significant genetic divergence from field populations	Monitor genetic drift and implement corrective measures
6	Health Risks from Infections	Increased vulnerability to bacterial, fungal, and pest infestations	Cleanliness, sterility, fresh foods, balanced diet

The following measures can be taken to minimize the risk of pathogenic infections in mosquito colonies, while preserving beneficial normal flora such as gut microbiota, which are essential for mosquito health and vector competence:

- Maintain hygiene in insectary rooms and rearing equipment
- Avoid overuse of antibiotics or disinfectants that could disrupt microbial balance
- Use sterilized larval food only in experimental setups that require aseptic conditions
- Monitor colonies for signs of bacterial or fungal outbreaks
- Periodically assess microbial profiles if feasible

### 4.6 Biosecurity and contamination prevention in mosquito colonies

Mosquito colonies can be compromised by pests and opportunistic microorganisms that reduce egg survival, larval development, or adult longevity. Strict cleanliness and routine sanitation in the insectary are essential to prevent pest infestations. Common threats include ants (including predatory ants), cockroaches/roaches, and small arthropods such as booklice (Psocoptera) and mites, which can damage egg stocks and contaminate rearing trays. Fungal growth is promoted by excess organic matter, overfeeding, stagnant water, and poor aeration; therefore, careful feed management, regular cleaning, and adequate ventilation are critical to maintain colony health. Standard prevention measures include physical barriers (sealed doors/windows, screened vents), strict cleaning schedules for trays/cages, prompt removal of dead larvae/adults, avoidance of feed accumulation, and routine disinfection of reusable tools. Quarantine of newly introduced field material, restricting movement between colony rooms, and maintaining detailed logs further reduce cross-contamination. These practices are consistent with widely used insectary manuals and biosafety/biosecurity guidance for mosquito rearing facilities (24, 36, 135, 146, 160).

## 5 Conclusion

Mosquitoes of the genera *Anopheles*, *Culex*, and *Aedes* remain among the most important vectors of human diseases, and a detailed understanding of their morphology and bionomics is fundamental for both ecological research and disease control. The establishment and long-term maintenance of laboratory colonies provide indispensable tools for studies on mosquito genetics, physiology, vector competence, and evaluation of novel control strategies. Recent advances in rearing techniques, blood-feeding methods, microbial management, and colony standardization have greatly enhanced the reliability of laboratory strains. However, challenges such as inbreeding depression, loss of genetic diversity, and microbial imbalance continue to threaten colony stability. Addressing these issues through the adoption of improved protocols, genetic management, and integration of emerging technologies will be crucial to sustain robust colonies that accurately represent field populations. Sustained investment in colony development, coupled with ecological insights, will not only strengthen basic entomological research but also accelerate translational approaches such as *Wolbachia*-based interventions, sterile insect technique (SIT), and other innovative vector control programs. Ultimately, bridging classical knowledge of mosquito biology with modern laboratory practices offers a pathway toward more effective and sustainable vector-borne disease control.
